# Hemodynamic Stability Between Spinal and General Anesthesia in Patient Undergoing Primary Total Knee Arthroplasty: A Retrospective Study

**DOI:** 10.1155/anrp/9045822

**Published:** 2025-12-03

**Authors:** Thomas Jeandin, Eric Albrecht, Jean-Benoit Rossel, Julien Wegrzyn, Matthieu Cachemaille

**Affiliations:** ^1^ Department of Anesthesia, Lausanne University Hospital and University of Lausanne, Lausanne, Switzerland, chuv.ch; ^2^ Centre for Primary Care and Public Health (Unisante), University of Lausanne, Lausanne, Switzerland, unil.ch; ^3^ Department of Orthopaedics and Traumatology, Lausanne University Hospital and University of Lausanne, Lausanne, Switzerland, chuv.ch; ^4^ Pain Clinic and Department of Anesthesia, La Tour Hospital, Meyrin, Switzerland, la-tour.ch

**Keywords:** general anesthesia, hemodynamic instability, knee arthroplasty, spinal anesthesia

## Abstract

**Background:**

Total knee arthroplasty (TKA) is a common surgical procedure that may be associated with blood loss. The aim of this study is to evaluate intraoperative hemodynamic stability during TKA under general (GA) or spinal anesthesia (SA).

**Methods:**

Any adult undergoing primary TKA under GA or SA was retrospectively selected over a 10‐year period and compared. The primary outcome was the presence of intraoperative hemodynamic instability, defined by starting a norepinephrine infusion, as the variation of the patient’s blood pressure exceeded 30% of its baseline value for more than 5 min. Secondary outcomes included intraoperative dose of ephedrine, phenylephrine, blood loss, and volume of fluid administered. Demographic and intraoperative anesthetic variables associated with norepinephrine use were entered in a multivariate logistic regression model.

**Results:**

The analysis included 1,441 patients; 59% received a SA. 3.6% of patients under SA required a norepinephrine infusion versus 10.4% under GA (*p* < 0.001). Ephedrine administered was lower in the SA group (mean dose 19 mg vs. 31 mg, *p* < 0.001), while phenylephrine was not statistically different (414 μg for SA and 481 μg for GA, *p* = 0.09). Intraoperative blood loss was identical in both groups (402 mL for SA and 415 mL for GA, *p* = 0.35), while mean intraoperative fluid income was higher in the GA group (862 mL vs. 725 mL, *p* < 0.001). Variables associated with norepinephrine use were GA and age, among others.

**Conclusions:**

GA requires more norepinephrine infusion compared to SA in patients during primary TKA, suggesting intraoperative hemodynamic stability is better preserved during neuraxial anesthesia.

## 1. Introduction

Total knee arthroplasty (TKA) is a very common surgical procedure in daily practice, and its number is constantly increasing due to the aging population [1]. It improves the quality of life of patients suffering from knee arthritis by relieving their joint pain and restoring their mobility [1]. This surgery is usually performed under general (GA) or spinal anesthesia (SA) in the absence of a clear consensus [1–8], while a myriad of studies have shown that SA reduces postoperative morbidity and mortality [1–7, 9–20].

Notably, GA has been associated with intraoperative hemodynamic instability for other orthopedic procedures such as lumbar spine surgery [21, 22]. Special attention to perioperative hemodynamic management in total knee and hip replacement surgeries under GA showed that hemodynamic stability is associated with less postoperative complications [23, 24]. Concerning TKA, peripheral nerve blocks have been reported to be correlated with a better stability than GA [25].

However, data on hemodynamic stability during TKA remain scarce, and to our knowledge, no study specifically compared hemodynamic stability between SA and GA for patients undergoing TKA, while several studies on hip fracture repair surgeries demonstrated that SA is associated with better hemodynamic stability, with fewer episodes of perioperative hypotension [26–28], shorter time duration spent in hypotension [26, 29], and less vasopressor administration [26, 27, 29].

Therefore, the aim of this study is to confirm the hypothesis that intraoperative hemodynamic stability is better preserved during SA when compared to GA.

## 2. Methods

Ethical approval for this study (Ethical Committee CER‐VD N8 2021‐00436) was provided by the Ethical Committee in Lausanne, Switzerland (Commission Cantonale d’Ethique de la Recherche sur l’Etre Humain, Chairperson Professor W. Pralong) on the 12th of April 2021. This manuscript adheres to the STrengthening the Reporting of OBservational studies in Epidemiology (STROBE) guidelines. The use of patients’ data was also approved by the ethical committee with waived consent.

### 2.1. Study Design

Data were retrospectively extracted from every anesthetic datasheet to be analyzed.

Baseline characteristics of patients extracted were age, sex, weight, height, body mass index, ASA class, anesthetic strategy, presence or absence of a femoral nerve block, presence or absence of a saphenous nerve block at the adductor canal, presence or absence of a femoral nerve catheter, and presence or absence of a local infiltration by the surgeon.

Intraoperative data extracted were norepinephrine infusion administration and maximal dosage of its infusion, ephedrine administration and total dosage, phenylephrine administration and total dosage, intraoperative fluid income, intraoperative blood loss, intravenous opioid administration, and total dosage.

### 2.2. Patient Selection

Inclusion criteria included patients ≥ 18 years of age undergoing primary TKA at the CHUV over a period of 10 years between the 1^st^ of October 2010 and the 31^st^ of October 2020.

Exclusion criteria included documented general research consent denied and other types of operation such as knee arthroplasty revision or reimplantation, partial revision of knee arthroplasty, and knee arthroplasty removal.

### 2.3. Outcomes

The primary outcome is the presence of hemodynamic instability, defined by a norepinephrine infusion started when the variation of the patient’s blood pressure exceeded 30% of its baseline value for more than 5 min. This definition implemented in our university hospital was used throughout the whole duration of our study and is documented on each anesthesia sheet.

Secondary outcomes are the maximal dosage of norepinephrine infusion when used, total intraoperative dosage of ephedrine and phenylephrine, volume of fluid administered, intraoperative blood loss, total intraoperative income/loss balance, and total intravenous dosage of opioids. More specifically, only total blood loss is reported and includes the amount of blood collected in the surgical aspiration; the number of surgical compresses impregnated with blood, which were also weighed; and blood loss coming from the operative field, which was estimated.

To facilitate data analysis and comparison, all opioid doses were converted to sufentanil equivalent doses with a conversion factor of 10 for fentanyl and 1000 for morphine.

### 2.4. Statistical Analyses

The SA group was compared with the GA group. Categorical values are described by their raw number and percentage, and a comparison is made using Pearson’s chi‐squared test. Continuous values are described by their mean and standard deviation, and a comparison is made using Student’s *t*‐test. The differences between both the groups are illustrated yearly between 2010 and 2020 for peripheral nerve block and surgical infiltration.

In the second phase, we used logistic regression models to explore if there is an association between some variables and the use of norepinephrine. We started with univariate logistic regressions with the following predictors: sex; age; BMI; ASA class; anesthetic strategy; administration of phenylephrine, ephedrine, and intraoperative intravenous opioid; interoperative fluid income; intraoperative blood loss; and finally, total intraoperative income/loss balance. We then built a multivariable logistic regression model, starting with variables such that *p* < 0.2 in the coefficient nullity test, and proceeded by backward elimination to select the most important factors.

Statistics were made using R software Version 4.0.5 (R Foundation for Statistical Computing, Vienna, Austria).

## 3. Results

**Figure 1:**
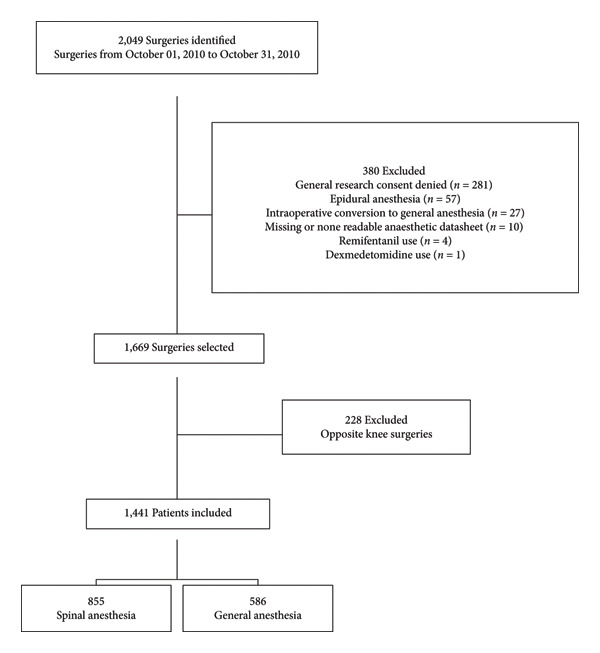
Patient selection.

We identified 2049 surgeries between October 1^st^ 2010, and October 31^st^ 2020, and 1,441 were finally selected and analyzed (Figure 1). 380 surgeries were excluded; 281 due to the absence of the patient’s general research consent, 57 due to another locoregional technique used (epidural anesthesia), 27 due to intraoperative conversion from SA to GA, 5 due to the addition of other pharmacological agents, remifentanil (4) and dexmedetomidine (1), and 10 due to missing or nonreadable anesthesia sheet. Finally, regarding patients who underwent TKA on both knees during the study period, only the first surgery was selected resulting in the exclusion of 228 procedures.

Baseline characteristics of all patients according to the anesthetic strategy are presented in Table 1. Both groups were similar except for a higher number of women in the GA group, 63.8% versus 56.5% (*p* = 0.006), and a year of age older in the GA group, 69 ± 11 versus 70 ± 10 years of age (*p* = 0.04).

**Table 1:** Baseline characteristics of all patients according to the anesthetic strategy.

	General anesthesia (*n* = 586, 40.7%)	Spinal anesthesia (*n* = 855, 59.3%)	Total (*n* = 1441, 100.0%)	*p* value
Sex				0.006
Female	374 (63.8)	483 (56.5)	857 (59.5)	
Male	212 (36.2)	372 (43.5)	584 (40.5)	
Age (years)	69 ± 11	70 ± 10	69 ± 10	0.04
Height (cm)	166 ± 10	167 ± 10	166 ± 10	0.14
Weight (kg)	82 ± 17	82 ± 17	82 ± 17	0.59
BMI (kg m^−2^)	30 ± 5	30 ± 6	30 ± 6	0.74
ASA Class				
I	25 (4.3)	47 (5.5)	72 (5.0)	0.53
II	396 (67.6)	549 (64.2)	945 (65.6)	
III	164 (28.0)	257 (30.1)	421 (29.2)	
IV	1 (0.2)	2 (0.2)	3 (0.2)	
Peripheral nerve block				
No	23 (3.9)	8 (0.9)	31 (2.2)	< 0.001
Yes	563 (96.1)	847 (99.1)	1410 (97.8)	
Surgical infiltration				
No	456 (77.8)	495 (57.9)	951 (66.0)	< 0.001
Yes	130 (22.2)	360 (42.1)	490 (34.0)	

*Note:* Data are presented either by the *n* (%) for categorical values (Pearson test) or by the mean ± SD for continuous values (Student test).

Abbreviation: BMI, body mass index.

**Figure 2:**
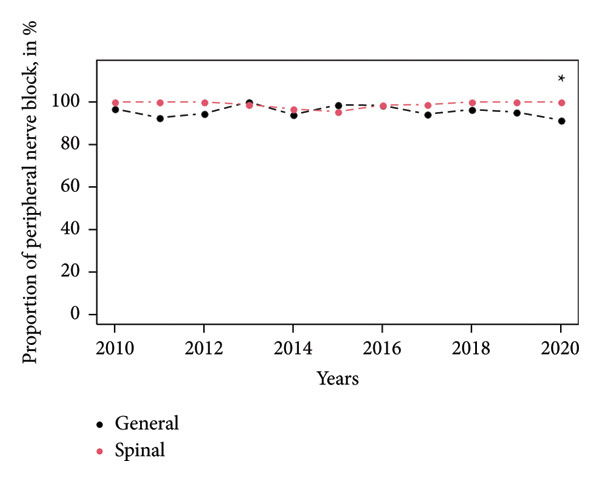
Peripheral nerve block from 2010 until 2020. ^∗^ *P*‐value  < 0.05. Data are presented by the mean for continuous values (Student test).

**Figure 3:**
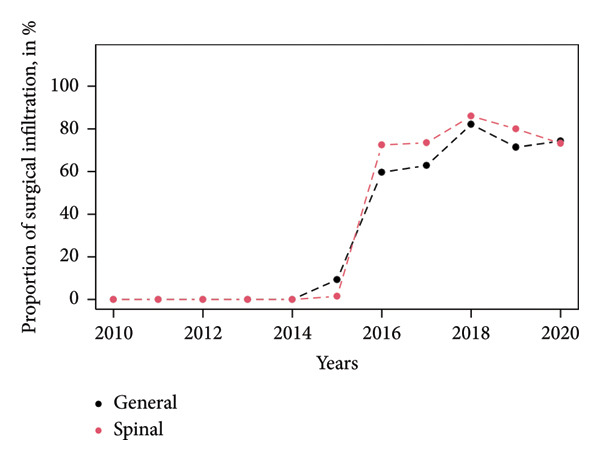
Surgical infiltration from 2010 until 2020 (no surgical infiltrations are reported from 2010 until 2014). Data are presented by the mean for continuous values (Student test).

Peripheral nerve blocks and surgical infiltrations were more often performed in the SA group, 99.1% versus 96.1% (*p* < 0.001) and 42.1% versus 22.2%, respectively (*p* < 0.001). During the study period, the number of peripheral nerve block performances was constant (Figure 2), and surgical infiltrations showed a decreased tendency lately (Figure 3).

Intraoperative data according to the anesthetic strategy are described in Table 2. Patients under SA required less often a norepinephrine infusion, 3.6% versus 10.4% (*p* < 0.001), while there was no significant difference between the mean transient highest dose in both the groups, 4.4 μg ± 2.4 versus ± 2.3, respectively (*p* = 0.98). If a vasopressor needed to be administered, it was more often performed in the GA group versus in the SA group for ephedrine, 75.8% versus 59.3% (*p* < 0.001), and for phenylephrine, 47.8% versus 29.7% (*p* < 0.001). Total dosage of ephedrine administered was lower in the SA group, 19 ± 14 mg versus 31 ± 19 mg (*p* < 0.001) as well as total dosage of phenylephrine, however, not statistically significant, 414 ± 496 μg versus 481 ± 412 μg (*p* = 0.09). Concerning the intraoperative volume status, intraoperative blood loss was identical in both groups, 402 ± 242 mL for SA and 415 ± 258 mL for GA (*p* = 0.35), while the GA group received more intraoperative fluid compared to the SA group, 862 ± 319 mL versus 725 ± 292 mL (*p* < 0.001). Intravenous opioids were less administered in the SA group, 7.3% versus 100% (*p* < 0.001), and if given, their total dosage was inferior, 8 ± 6 μg versus 47 ± 19 μg (*p* < 0.001).

**Table 2:** Intraoperative data according to the anesthetic strategy.

	General anesthesia (*n* = 586, 40.7%)	Spinal anasthesia (*n* = 855, 59.3%)	Total (*n* = 1441, 100.0%)	*p* value
Primary outcome				
Norepinephrine infusion				
No	525 (89.6)	824 (96.4)	1349 (93.6)	< 0.001
Yes	61 (10.4)	31 (3.6)	92 (6.4)	
Secondary outcomes				
Maximum dosage of norepinephrine infusion (μg min^−1^) (ml h^−1^)	4 ± 2	4 ± 2	4 ± 2	0.98
Total ephedrine dosage (mg)	31 ± 19	19 ± 14	25 ± 18	< 0.001
Total phenylephrine dosage (μg)	481 ± 412	414 ± 496	449 ± 455	0.09
Intraoperative fluid income (ml)	862 ± 319	725 ± 292	781 ± 311	< 0.001
Intraoperative blood loss (ml)	415 ± 258	402 ± 242	407 ± 249	0.35
Total intraoperative income/loss balance (ml)	453 ± 350	332 ± 293	382 ± 323	< 0.001
Total intravenous opioid dosage (μg)	47 ± 19	8 ± 6	43 ± 21	< 0.001

*Note:* Data are presented either by the *n* (%) for categorical values (Pearson test) or by the mean ± SD for continuous values (Student test).

Univariate logistic regressions were used to measure the association between each variable and norepinephrine administration for all patients (Table 3). Intraoperative variables that favor the use of norepinephrine are GA (OR: 3.09, 95% CI: 1.98–4.82, *p* < 0.001), phenylephrine administration (OR: 4.78, 95% CI: 2.99–7.63, *p* < 0.001), and intravenous opioid administration (OR: 3.16, 95% CI: 1.99–5.02, *p* < 0.001). Patient characteristics that favor the use of norepinephrine are ASA III/IV (OR: 6.28, 95% CI: 3.96–9.95, *p* < 0.001) and age (OR: 1.06, 95% CI: 1.03–1.08, *p* < 0.001).

**Table 3:** Univariate logistic regression model to predict the use of norepinephrine.

	Odds ratio	*p* value
Sex: male	0.89 (0.58–1.38)	0.62
Age	1.06 (1.03–1.08)	0.001
BMI	1.03 (0.99–1.07)	0.11
ASA Class III/IV	6.28 (3.96–9.95)	0.001
Anesthetic strategy: GA	3.09 (1.98–4.82)	< 0.001
Phenylephrine administration	4.78 (2.99–7.63)	< 0.001
Ephedrine administration	0.75 (0.49–1.16)	0.20
Intravenous opioid administration	3.16 (1.99–5.02)	< 0.001
Intraoperative fluid income	1.00 (1.00–1.00)	0.40
Intraoperative blood loss	1.00 (1.00–1.00)	0.11
Total intraoperative income/loss balance	1.00 (1.00–1.00)	0.44

*Note:* Data are presented by the odds ratio (95% confidence intervals) (Wald test).

Abbreviation: BMI, body mass index; GA, general anesthesia.

A multivariable logistic regression model was used to select the variables that are the most associated with norepinephrine administration (Table 4). Intraoperative variables that were associated with norepinephrine use are GA (AOR: 2.87, 95% CI: 1.77–4.66, *p* < 0.001) and phenylephrine administration (AOR: 3.36, 95% CI: 2.05–5.51, *p* < 0.001). Patient characteristics associated with the use of norepinephrine are ASA Class III/IV (AOR: 5.89, 95% CI: 3.64–9.54, *p* < 0.001) and age (AOR: 1.05, 95% CI: 1.02–1.07, *p* < 0.001).

**Table 4:** Multivariable logistic regression model to predict the use of norepinephrine, including selected variables from Table 3.

	Adjusted odds ratio	*p* value
Age	1.05 (1.02–1.07)	< 0.001
ASA Class III/IV	5.89 (3.64–9.54)	< 0.001
Anesthetic strategy: GA	2.87 (1.77–4.66)	< 0.001
Phenylephrine administration	3.36 (2.05–5.51)	< 0.001

*Note:* Data are presented by the adjusted odds ratio (95% confidence intervals) (Wald test).

Abbreviation: GA, general anesthesia.

## 4. Discussion

This retrospective study shows that patients undergoing a primary TKA under SA require significantly less often a norepinephrine infusion compared to patients under GA. This suggests that intraoperative hemodynamic stability is better preserved during SA.

Benefits of SA regarding hemodynamic stability were already reported during hip fracture repair surgeries, by Simonin et al. in elderly patients, mostly ASA Class II, with fewer episodes of perioperative hypotension, 20.3% versus 58.9% in the open surgery group and 22.6% versus 62.2% in the percutaneous group, respectively (*p* < 0.001) [26]; by Biboulet et al. in elderly patients with cardiovascular comorbidities, median of 0 (range: 0–6) versus 11.5 (range: 1–25) for GA with propofol and 10 (range 1–23) for GA with sevoflurane (*p* < 0.001) [27]; and by Li et al., 78.3% versus 31.6% in the regional anesthesia group (*p* < 0.001) [28]. Furthermore, shorter perioperative time spent in hypotension was described by Simonin et al., median of 0 min (range: 0–12.0) for SA versus 23.5 min (range: 9–35.5) for GA (*p* < 0.01) [26], and Messina et al., focusing on hemodynamic monitoring in elderly patients, mostly ASA Class III, showed a longer overall time percentage spent over hypotension, 69.7% for SA versus 38.9% for GA (*p* < 0.001) [29].

In our study, although norepinephrine infusion’s frequency was superior in the GA group, no significant difference was observed regarding the transient highest dosage administered. The hypothesis is that the management of hemodynamic instability is similar in both groups by requiring the same loading dose of vasopressor followed by a relatively short duration of infusion. Although we were not able to measure the total dose of norepinephrine delivered to our patients, Simonin et al. found no difference in their study [26].

In our results, vasopressors were significantly less often administered in the SA group for both ephedrine and phenylephrine, which supports the primary outcome results tendency. Interestingly, these findings were also observed in three other studies during hip surgeries [26, 27, 29]. Moreover, when vasopressors were injected, we found that the total dose of ephedrine was inferior in the SA group, although not statistically significant for the phenylephrine. Our results conquer with studies from Biboulet et al. and Messina et al., who also reported a mean difference of ephedrine administered between patients under SA versus GA, either under propofol or sevoflurane [27, 29]. Regarding the nonsignificant difference in phenylephrine dosage between the groups, during SA, the sympathetic nerve block decreases peripheral vascular resistance, creating vasodilatation, thus resulting in hypotension. The first reflex, without a bradycardia state, is to increase peripheral resistance by using an alpha‐1 agonist. Therefore, phenylephrine is preferred compared with ephedrine, and that may explain the nonsignificant total dosage found between the groups.

A superior positive total intraoperative income/loss balance was observed under GA with similar blood loss but a higher fluid administration. Similar results were reported by Messina et al. during hip fracture surgeries with a superior mean fluid administration, 1039 ± 330 mL for SA versus 1338 ± 285 mL for GA (*p* < 0.05) [29]. As patients in our study were selected under a planned surgery, without a preemptive fracture, the shorter fasting time may result in a lower fluid administration. Finally, an inferior total perioperative income/loss balance also suggests that SA may give a better hemodynamic stability. However, future prospective studies should standardize fluid protocols to minimize bias.

Intravenous opioids were less administered in the SA group, as they were used to potentiate the sedation or the analgesia at the end of the surgery for a small number of patients compared to the GA group, where they were given more freely along the whole surgery. Although statistically significant and associated with norepinephrine use in the univariate analysis, they were not linked to the use of norepinephrine in the multivariate analysis. With the current design of our study, as GA implies their use, we believe it might be a confounding bias and cannot conclude that opioids might contribute to hemodynamic instability on their own. To explore such a question, further research projects should be made with an alternative design focusing on opioid administration.

**Figure 4:**
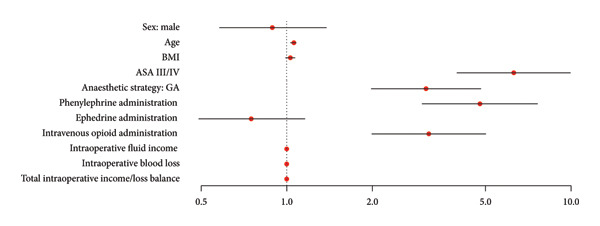
Visual representation of Table 3 illustrated with a forest plot: univariate logistic regression model to predict the use of norepinephrine. Data are presented by the odds ratio (95% confidence intervals) (Wald test), logarithmic scale. BMI, body mass index; GA, general anesthesia.

**Figure 5:**
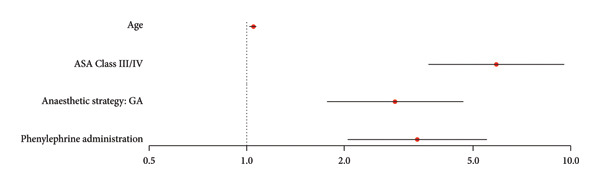
Visual representation of Table 4 illustrated with a forest plot: multivariable logistic regression model to predict the use of norepinephrine including the selected variables from Table 3. Data are presented by the adjusted odds ratio (95% confidence intervals) (Wald test), logarithmic scale. GA, general anesthesia.

To highlight parameters that could be associated with the use of norepinephrine, a univariate logistic regression model was used followed by a multivariable model, Table 3 and Figure 4 and Table 4 and Figure 5, respectively. Finally, age, ASA Class III/IV, GA, and phenylephrine administration were linked to the use of norepinephrine.

An older patient with cardiac comorbidities is more likely to receive norepinephrine infusion to increase peripheral vascular resistance and inotropism. Patients who received phenylephrine, most of the time, already received ephedrine first. The next step, during a prolonged hypotension state, despite a previous ephedrine and phenylephrine intake, is to administer norepinephrine. Consequently, patients who received phenylephrine were more supposed to receive norepinephrine. Interestingly ephedrine administration was not associated with norepinephrine infusion. Given the explanation that ephedrine in our hospital is administered as a first‐line and norepinephrine as a third‐line treatment during unstable hemodynamic states, both variables may be uncorrelated.

The first major limitation of this research project is its retrospective design, which may be a bias in the quality of the data collected. For example, confounding bias, such as different sources of intraoperative bleeding rate, has not been considered. On the other hand, the potential link between intraoperative instability and postoperative organ injury was not monitored and would warrant a prospective evaluation of AKI/myocardial injury in a future study.

Second, we must keep in mind that the choice of the anesthetic strategy was made according to nonstandardized criteria coming from the anesthesiologist in charge, which could limit the interpretation of our results. Comorbidities such as intracranial hypertension, severe aortic stenosis, or obstructive hypertrophic cardiomyopathy are specific contraindications to SA and require most of the time a GA. Patients with these pathologies are more fragile and may be prone to more hemodynamic instability. While ASA classification broadly captures cardiovascular risk, unmeasured variability in individual comorbidities, such as hypertension, may influence hemodynamic outcomes.

Third, we used an indirect method to determine hemodynamic instability through the initiation of a norepinephrine infusion. Applying the direct definition of hemodynamic instability by measuring the MAP continuously could provide different information and accuracy. MAP monitoring gold standard is measured by an invasive arterial catheter, which was not used in our study due to the limited risk/benefice ratio of such a device for primary TKA surgeries. Further developed and recently validated, MAP interpretation via a continuous noninvasive arterial pressure (CNAP) measurement might be a new technique to use for further investigations [30]. Furthermore, evaluating perfusion efficacy with a microcirculation monitoring, using a sublingual video microscopy, might also be of interest. Finally, real‐time computerized registration of perioperative anesthetic data including vital parameters, drug administration, and surgical phases could help to collect data more precisely and help in research development.

Further prospective studies considering these previous suggestions could help to understand and prevent hemodynamic instabilities and therefore the negative consequences to patients’ health.

## 5. Conclusion

GA is correlated with a higher rate of norepinephrine infusion compared to SA in patients undergoing primary TKA in our hospital, suggesting intraoperative hemodynamic stability is better preserved during SA.

## Disclosure

Preliminary data for this study were presented as a poster presentation at the European Society of Regional Anesthesia Virtual Congress, 8–10 September 2021, and at the Swiss Anesthesia Annual Meeting, on the 28th of October 2021.

## Conflicts of Interest

EA or his institution has received educational, honoraria or research funding from the Swiss National Science Foundation, the Swiss Academy for Research in Anesthesia, B. Braun Medical AG Switzerland, Sintetica Ltd UK, and MSD AG Switzerland. The other authors declare no conflicts of interest.

## Author Contributions

Thomas Jeandin: data curation, formal analysis, investigation, and writing–original draft. Eric Albrecht: methodology, conceptualization, funding acquisition, supervision, and writing–review and editing. Jean‐Benoit Rossel: formal analysis and writing–review and editing. Julien Wegrzyn: methodology and writing–review and editing. Matthieu Cachemaille: conceptualization, methodology, data curation, formal analysis, supervision, writing–review and editing, and project administration.

## Funding

This work was supported by departmental funding (Department of Anesthesia, University Hospital of Lausanne, University of Lausanne, Lausanne, Switzerland).

## Supporting Information

Supporting Information: Reporting guidelines STROBE statement.

## Supporting information


**Supporting Information** Additional supporting information can be found online in the Supporting Information section.

## Data Availability

The data that support the findings of this study are available from the corresponding author upon reasonable request.
